# Sudden Respiratory Failure after a Coughing Paroxysm

**DOI:** 10.7759/cureus.6221

**Published:** 2019-11-23

**Authors:** André Rosa Alexandre, Sérgio Santos Pinto

**Affiliations:** 1 Internal Medicine and Intensive Care Department, Hospital Da Luz, Lisboa, PRT; 2 General Emergency Department, Hospital Beatriz Ângelo, Loures, PRT

**Keywords:** diaphragmatic hernia, respiratory failure, emergency medicine

## Abstract

We report a case of an 85-year-old female, previously demented and dependent on others, who presented to the emergency department with sudden dyspnea following a coughing paroxysm. She was polypneic and with a diminished vesicular murmur at pulmonary auscultation but with audible bowel sounds in the right hemithorax. Arterial blood gases showed hypoxemic respiratory failure but the additional blood work was unremarkable. A thoracic radiograph suggested the presence of small bowel on the thoracic cavity. A thoracic computed tomography confirmed the diagnosis of an anterior right giant paracardiac transdiaphragmatic hernia of small bowel through the foramen of Morgagni with secondary passive pulmonary atelectasis. A posterior left transhiatal gastric hernia was also found. She was treated conservatively with nasogastric intubation and discharged home two days later, asymptomatic and without respiratory failure. Spontaneous diaphragmatic hernias are extremely rare, non-traumatic surgical emergencies, almost invariably requiring surgical correction. This case shows that a conservative approach is an alternative in selected patients.

## Introduction

Spontaneous diaphragmatic hernias (SDHs) are extremely rare, non-traumatic surgical emergencies (<1% of all diaphragmatic hernias). There are only around 30 reports of this entity in the literature [1-3]. An SDH originates from a lesion to the diaphragm induced by a sudden increase in intra-abdominal pressure, which leads to the extension of intra-abdominal contents into the thoracic cavity through the resulting diaphragmatic defect. The more frequently reported causes of the rise in intra-abdominal pressure leading to SDH are physical exercise, labor and delivery, coughing, vomiting, and defecation [4].

## Case presentation

An 85-year-old female, dependent on others because of advanced dementia, and with a past medical history of hypothyroidism and peptic ulcer disease, presented to the emergency department (ED) with complaints of dyspnea, productive cough, and fever with 48 hours of evolution. She was diagnosed with community-acquired pneumonia and discharged home with amoxicillin-clavulanate. That decision was based on the absence of clinical criteria of gravity such as respiratory failure or distress.

After four days, she returned to the ED with a sudden worsening of dyspnea following a coughing paroxysm. On a physical examination, she was hemodynamically stable but polypneic, with a diminished vesicular murmur at pulmonary auscultation and with audible bowel sounds in the right hemithorax. The rest of the physical examination was unremarkable.

Arterial blood gases showed hypoxemic respiratory failure. Complete blood count, renal function, serum ionogram, and C-reactive protein were within the normal range.

**Figure 1:**
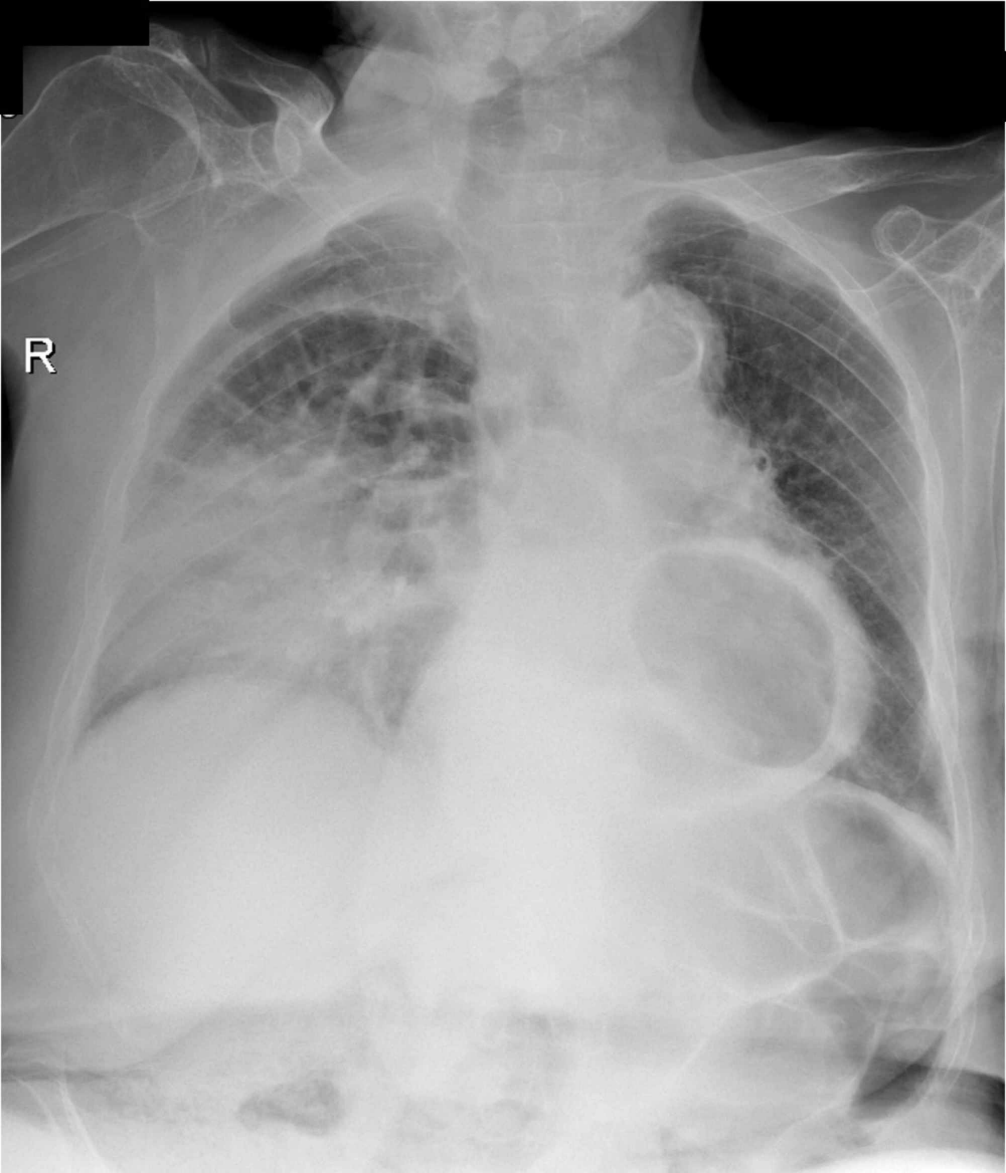
Chest radiograph suggesting the presence of the small bowel inside the thoracic cavity, collapsing the right lung

**Figure 2:**
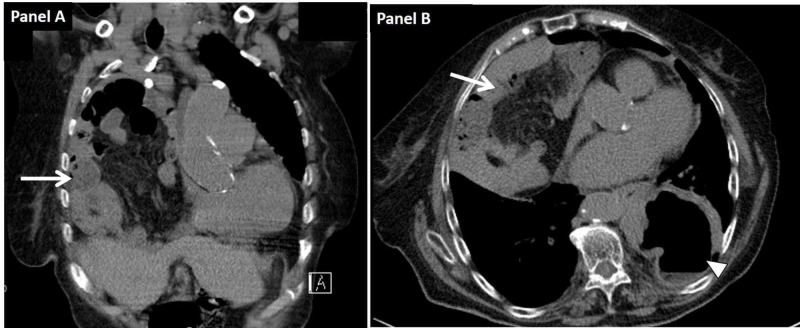
Thoracic computed tomography showing two giant transdiaphragmatic hernias Panel A: Coronal view; Panel B: Transversal view; Arrow: anterior giant paracardiac transdiaphragmatic hernia; Arrowhead: posterior transhiatal gastric hernia

A thoracic radiograph (Figure 1) suggested the presence of the small bowel inside the thoracic cavity, which was confirmed by a thoracic computed tomography (Figure 2). The diagnosis of an anterior right giant para-cardiac transdiaphragmatic hernia of the small bowel through the foramen of Morgagni (Figures 2A-2B; arrow) with secondary passive pulmonary atelectasis and of a posterior left transhiatal gastric hernia (Figure 2B; arrowhead) was made.

The general surgery team evaluated the patient, and she was treated conservatively with nasogastric intubation. The evolution was excellent and, after two days, she was discharged home asymptomatic and without respiratory failure.

## Discussion

The diagnosis of SDH requires high clinical suspicion and depends on thoracic imaging. There are some red flags that should not be missed to diagnose this entity: the known presence of a previous diaphragmatic defect, dyspnea following a sudden intra-abdominal pressure increasing the maneuvre or dyspnea with vomiting [5-6].

This patient’s presentation suggests that the coughing paroxysm was the plausible cause of the increase in intra-abdominal pressure leading to the anterior right giant paracardiac transdiaphragmatic hernia of the small bowel through the foramen of Morgagni.

The most remarkable findings on physical examination are the absence of breath sounds and the presence of bowel sounds in the thoracic cavity [6]. This patient presented both signs.

Chest radiograph and computed tomography are the most accurate imaging techniques for an SDH diagnosis and are readily available. Yet, it is estimated that 66% of SDHs are missed on initial presentation [7]. Point-of-care ultrasound is another imaging option to diagnose an SDH and its recent dissemination may contribute to decreasing the rate of diagnosis failure. Typical findings of an SDH on ultrasound include the direct visualization of the diaphragmatic defects, the abolishment of pleural line visualization by the interposition of hernial content, and the visualization of omental vessels by the use of color Doppler [8].

The identification of an SDH should not be delayed because of its life-threatening potential. Early diagnosis can prevent complications, as bowel strangulation or perforation and pulmonary or vascular compression [6].

Although surgical repair is the mainstay of SDH treatment, nasogastric tube intubation and decompression can relieve symptoms. This case shows that a conservative approach can be an acceptable option for patients with a prohibitive surgical risk as exemplified by this case [2,6].

## Conclusions

SDHs are rare causes of respiratory failure but should be considered in the appropriate context. The prompt identification of an SHD is essential to prevent life-threatening complications. Surgical treatment is almost always the indicated therapy, but in selected cases, a conservative approach with nasogastric tube intubation and decompression may suffice.
